# Sustainable Geopolymer Mortars from Ceramic Sanitaryware Waste: Impact of Curing Methods on Mechanical and Thermal Behavior

**DOI:** 10.3390/ma19112214

**Published:** 2026-05-24

**Authors:** Rim Benkabou, Abir Rezzoug, Kada Ayed, Aissa Asroun, Zouaoui R. Harrat, Mohammed Chatbi, Ercan Işık, Fatih Avcil, Marijana Hadzima-Nyarko

**Affiliations:** 1 Laboratory of Civil Engineering and Environment (LGCE), Department of Civil Engineering and Public Works, Djillali Liabès University, Sidi Bel Abbès 22000, Algeria; 2 Engineering Materials Laboratory (Labmat), National Polytechnic School Maurice Audin (ENPO-MA), El M’Naouer, Oran 31000, Algeria; 3 Laboratoire des Structures et Matériaux Avancés dans le Génie Civil et Travaux Publics, University of Djillali Liabes, Sidi Bel Abbès 22000, Algeria; 4 Department of Public Works, University Mouloud Mammeri, Tizi Ouzou 15000, Algeria; 5 Department of Civil Engineering, Bitlis Eren University, Bitlis 13100, Türkiye; 6 Faculty of Civil Engineering and Architecture, Josip Juraj Strossmayer University of Osijek, 31000 Osijek, Croatia

**Keywords:** ceramic sanitaryware waste, compressive strength, curing method, geopolymer mortar, microstructure

## Abstract

**Highlights:**

- Curing temperature and duration significantly affect mechanical performance.
- After 800 °C exposure, lower curing duration improves residual strength retention.
- Direct curing yields higher compressive strength than delayed curing.

**Abstract:**

This study investigates the influence of curing conditions on mechanical performance, residual strength after high-temperature exposure, and microstructural evolution of geopolymer mortars based on ceramic sanitaryware waste (CSW). Direct and delayed thermal curing regimes were applied at 60 °C and 80 °C for 48 h and 72 h. The fresh mixtures exhibited adequate workability with a flow diameter of 21 cm, indicating suitable consistency for casting. Results show that direct curing consistently enhances compressive strength, reaching 30.97 MPa at 80 °C for 72 h, compared with 15.88 MPa under delayed curing. Increasing curing temperature and duration improved early-age mechanical performance, particularly under direct curing conditions. After exposure to 800 °C, directly cured specimens retained higher residual compressive strength, with an improvement of approximately 6.6% compared with delayed-cured specimens. Microstructural characterization using scanning electron microscopy coupled with energy-dispersive spectroscopy and X-ray diffraction supported the observed mechanical trends under different curing conditions. The findings highlight the role of curing strategy in optimizing CSW-based geopolymer mortars for construction applications where mechanical performance and high-temperature resistance are required.

## 1. Introduction

The decarbonization of the construction sector has become an urgent global priority, as Portland cement production is responsible for significant CO_2_ emissions, estimated at approximately 0.5–0.95 tons of CO_2_ per ton of cement produced, in addition to intensive raw material and energy consumption [1,2]. In parallel, large quantities of construction and demolition waste, particularly ceramic sanitaryware waste (CSW), are continuously generated worldwide [3]. This accumulation is mainly attributed to the ongoing production of ceramic rejects and demolition activities, together with the limited recycling pathways available for vitrified ceramic materials. Owing to their final fired nature and the absence of efficient recycling routes, ceramic wastes are typically not reintroduced into conventional production cycles and are therefore disposed of in landfills, leading to long-term accumulation [4,5]. For example, the Algerian ceramic industry exceeds 200 million m^2^/year of production, further contributing to the continuous increase in ceramic waste generation for approximately 5–15% of total ceramic production. According to the the National Waste Information System (SNID), construction and demolition waste streams continue to increase steadily, reinforcing the environmental pressure associated with ceramic waste disposal [6].

These environmental challenges have encouraged the development of sustainable alternatives based on waste valorization strategies [7]. Geopolymers, which are synthesized through the alkaline activation of aluminosilicate precursors, have been widely recognized as eco-efficient construction materials. During geopolymerization, three-dimensional sodium–aluminosilicate are formed networks consisting of Si–O–Al–O bonds, resulting in materials that exhibit several advantageous properties, including high early-age strength, reduced carbon emissions, and excellent thermal stability [8]. Considerable research has been conducted on various precursor systems, including fly ash [9], ground granulated blast furnace slag [10], metakaolin [11], and other industrial by-products [12], demonstrating their potential for sustainable construction applications. Among these materials, increasing attention has been directed toward ceramic waste, particularly CSW, owing to its high silica and alumina contents, thermal stability, and wide availability from industrial and demolition sources [13].

Previous studies have demonstrated that ceramic waste can be successfully incorporated into geopolymer systems, resulting in satisfactory mechanical strength and durability under appropriate mix designs [14]. For instance, Milica Vasicć et al. [15] reported that ceramic waste could be successfully utilized to produce dense and mechanically stable geopolymer. Similarly, Rezzoug et al. [6] investigated CSW as a sole precursor under different curing temperatures and activator molarities, and reported that while higher molarity enhances early-age strength, it may become detrimental at later ages due to microstructural instability and reduced long-term performance. In addition, Bayer Öztürk et al. [16] examined the influence of water-to-binder ratio, activator molarity, and curing temperature, and demonstrated that a lower water-to-binder ratio of 0.45 resulted in higher compressive strength compared to 0.5, while increasing curing temperature significantly improved mechanical performance.

Furthermore, improved compressive strength and enhanced thermal behavior have been reported when ceramic-derived precursors were adequately activated [4,5,17]. Nevertheless, Fořt et al. [18] reported that CSW generally exhibits lower reactivity compared with fly ash or slag because of its partially crystalline and vitrified structure, which limits dissolution and geopolymer gel formation. Consequently, its performance was strongly governed by processing parameters.

The properties of geopolymer systems were controlled by multiple interacting factors, including precursor chemistry, amorphous content, particle fineness, activator composition, liquid-to-solid ratio, and curing conditions [19,20,21,22]. Among these parameters, curing conditions have received considerable attention in ceramic waste-based geopolymer systems due to their strong influence on geopolymerization kinetics, microstructural evolution, and mechanical performance. Most reported studies have focused on curing temperatures in the range of 40–100 °C, demonstrating that elevated curing temperatures accelerate dissolution and polycondensation reactions, thereby improving early-age strength development, particularly within the first 24 h of curing [6,16,23,24,25,26]. Consequently, several accelerated curing approaches have been explored to further enhance geopolymer reaction kinetics and reduce processing time. In addition to conventional oven curing, alternative techniques such as microwave curing and electrical curing have also been investigated. Microwave curing has been reported to promote rapid and relatively uniform internal heating, whereas electrical curing generates heat through ionic conduction within fresh geopolymer mixtures, accelerating early-age reaction development and strength gain [27]. Although these curing approaches have shown promising results, particularly for precast applications, their implementation in ceramic waste-based geopolymer systems remains relatively limited [1,28].

However, most existing studies have primarily considered short-term thermal curing of typically 24 h [29,30,31], while the influence of extended curing durations (48 h and 72 h) and curing pathway (direct versus delayed thermal activation) remains comparatively less explored. In particular, only limited studies have investigated delayed thermal curing approaches in geopolymer systems. For example, preliminary investigations on ceramic and brick waste-based geopolymers demonstrated that an initial ambient curing stage prior to thermal treatment significantly improved compressive strength compared with immediate heat curing, owing to better moisture redistribution and progressive gel development before thermal activation [32,33]. Similar observations were also reported in geopolymer mortar systems incorporating ground bottom ash [34], where specimens subjected to delayed thermal curing exhibited higher compressive strength than those directly exposed to heat curing immediately after casting. These findings suggest that the initial curing stage prior to thermal treatment may strongly influence geopolymer network formation and microstructural densification.

Moreover, previous investigations on metakaolin-based geopolymers demonstrated that curing regime plays a critical role in determining geopolymer structure and performance [35]. Moderate thermal curing temperatures combined with suitable curing durations were shown to induce strength development, whereas excessive temperatures or inappropriate curing durations could induce microstructural deterioration and reduced performance [36]. Such observations further indicate that the response to curing conditions strongly depends on precursor characteristics and geopolymer reaction mechanisms.

The influence of delayed thermal activation in geopolymer based-CSW may become even more critical for controlling early geopolymer network formation. Nevertheless, the combined effects of delayed curing strategy and extended thermal curing durations on CSW-based geopolymer mortars have not yet been systematically investigated.

Addressing these gaps is considered essential for improving the understanding of early-age reaction development and optimizing processing conditions for CSW-based geopolymer mortars. In the present study, the influence of heat curing pathway on the mechanical performance, residual mechanical behavior after thermal exposure, and microstructural evolution of CSW-based geopolymer mortars was investigated using scanning electron microscopy coupled with energy-dispersive spectroscopy (SEM/EDS) and X-ray diffraction (XRD) analysis. The following two curing strategies are considered: direct thermal curing (immediate oven exposure after casting) and delayed thermal curing (24 h ambient storage prior to thermal treatment). Both regimes are applied at 60 °C and 80 °C for 48 h and 72 h, while activator composition and liquid-to-solid ratio were kept constant.

## 2. Materials and Methods

### 2.1. Materials

**Figure 1:**
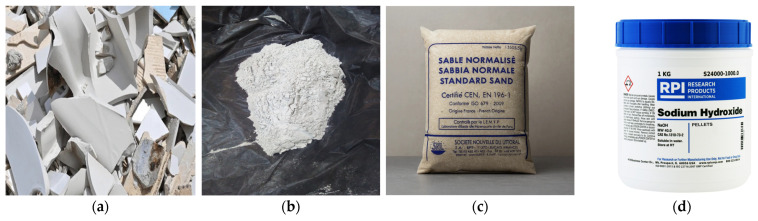
Materials used for mixtures preparation: (**a**) Solid ceramic sanitaryware waste; (**b**) Crushed ceramic sanitaryware waste; (**c**) Standard sand; (**d**) Sodium hydroxide.

The materials used for geopolymer production were collected from a ceramic factory in Tlemcen (Remchi, Algeria). The precursor consisted of ceramic sanitaryware waste (CSW) as illustrated in Figure 1a. A preliminary manual sorting was first performed to remove impurities and non-ceramic residues. The selected CSW was then manually pre-fragmented and subsequently crushed using a jaw crusher. The crushed material was ground in a planetary ball mill BB27 model (E2M2, Aubergenville, France) operating at 60 rpm (900 rotations per 15 min) for 3 h to obtain fine powder (Figure 1b). The resulting material was sieved through a 63 µm sieve, and the fraction passing the sieve was used for geopolymer preparation.

Standard silica sand [37] as shown in Figure 1c was used as fine aggregate in accordance with established mortar preparation standards. The alkaline activator (Figure 1d) consisted solely of a sodium hydroxide (NaOH) solution. Analytical-grade NaOH pellets (98% purity) were dissolved in distilled water to obtain a 10 M solution. The solution was prepared 24 h prior to mixing to ensure complete dissolution and thermal equilibration, in accordance with ASTM E200-16 [38]. The liquid-to-binder (L/B) ratio was fixed at 0.45 for all mixtures. The selected NaOH concentration and L/B ratio were based on previous studies on ceramic waste-based geopolymer systems [6]. The liquid-to-binder (L/B) ratio was fixed at 0.45 for all mixtures. The selected NaOH concentration and L/B ratio were based on previous studies on ceramic waste-based geopolymer systems.

### 2.2. Sample Preparation and Geopolymer Mortar Characterization

The geopolymer mortar mixtures were prepared by first mixing the CSW powder with the NaOH solution from Controls Group (Milan, Italy) using a mechanical mixer for 2 min. Standard silica sand was then gradually added, and mixing was continued for an additional 2 min 30 s, resulting in a total mixing time of 4 min 30 s to obtain a homogeneous mortar mixture. The fresh mortar was cast into 40 mm × 40 mm × 40 mm cubic molds and compacted using a vibrating mortar table (Controls Group, Milan, Italy) to minimize entrapped air. Immediately after casting, the mold surfaces were covered with a plastic film to prevent moisture loss and water evaporation.

Two curing protocols were applied to evaluate the influence of curing conditions on the geopolymerization process. The first protocol, referred to as direct thermal curing, consisted of placing the specimens directly in a controlled curing oven (Controls Group, Italy) after casting. The second protocol, designated as delayed thermal curing, involved storing the specimens under laboratory conditions at approximately 20 °C for 24 h prior to thermal curing. For both curing approaches, the specimens were then cured at temperatures of 60 °C and 80 °C for durations of 48 h and 72 h, resulting in eight different curing regimes. After curing, the specimens were demolded and stored under laboratory conditions until the testing age of 28 days. All mechanical tests were performed on triplicate specimens, and the reported results correspond to the average values of three measurements. The mixture proportions and specimen designations are summarized in Table 1.

Compressive strength tests were performed using a hydraulic testing machine (Advantest 9, (Controls Group, Italy) under a constant loading rate of 0.1 kN/s, in accordance with EN 196-1 [37] standard procedures for mortar testing.

To evaluate the residual mechanical performance, specimens were heated to 800 °C for 1 h in a furnace (Protherm Furnaces, Ankara, Turkey) at a heating rate of 5 °C/min and then cooled naturally to room temperature, after which the compressive strength was measured to determine strength retention following thermal exposure.

In addition, scanning electron microscopy (SEM/EDX) observations were carried out using a Hitachi TM1000 (Hitachi High-Tech, Tokyo, Japan) microscope to examine the microstructural features of the samples.

**Figure 2:**
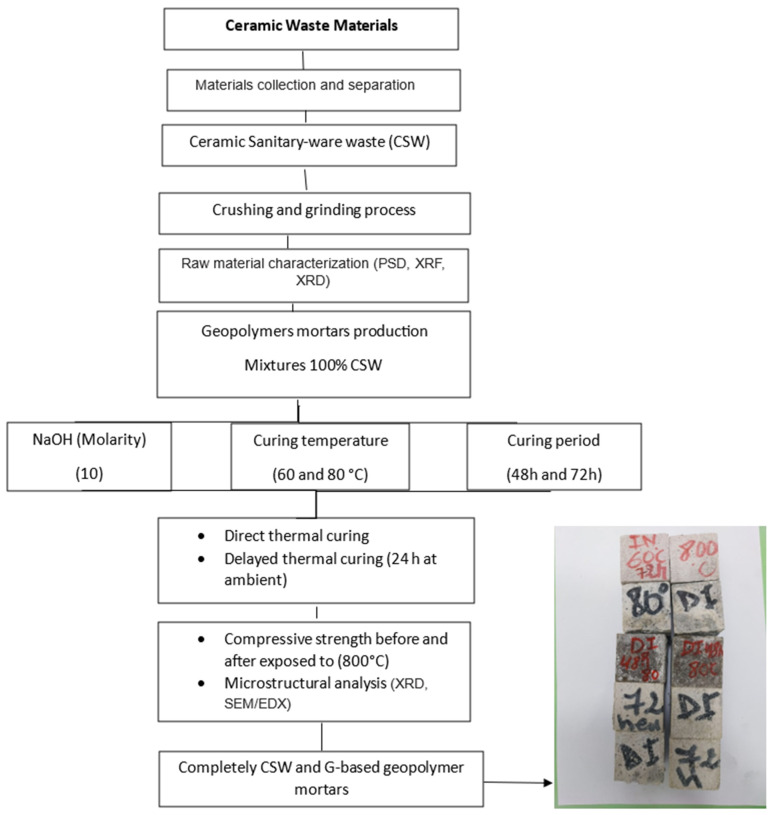
Flowchart of the study.

A schematic overview of the experimental procedure is presented in the flowchart shown in Figure 2.

## 3. Results and Discussion

### 3.1. Material Characterization

The physical and chemical properties of the ceramic sanitaryware waste (CSW) used in this study are summarized in Table 2. The material was mainly composed of silica (69.56 wt.% SiO_2_) and alumina (22.61 wt.% Al_2_O_3_), with a specific gravity of 2.6. The specific surface area was measured at 3500 cm^2^/g using an automatic BSA1 Blaine air permeability apparatus (Global Instruments Corporation, Nashik, India) in accordance with ASTM C204-18e01 standards [39]. The obtained value was consistent with those reported for similar ceramic waste materials in previous studies [14]. The oxide composition was determined by X-ray fluorescence (XRF) analysis using a (Bruker, Karlsdorf, Germany) S8 Tiger spectrometer model. Instrument calibration and method validation were performed using certified reference materials.

**Figure 3:**
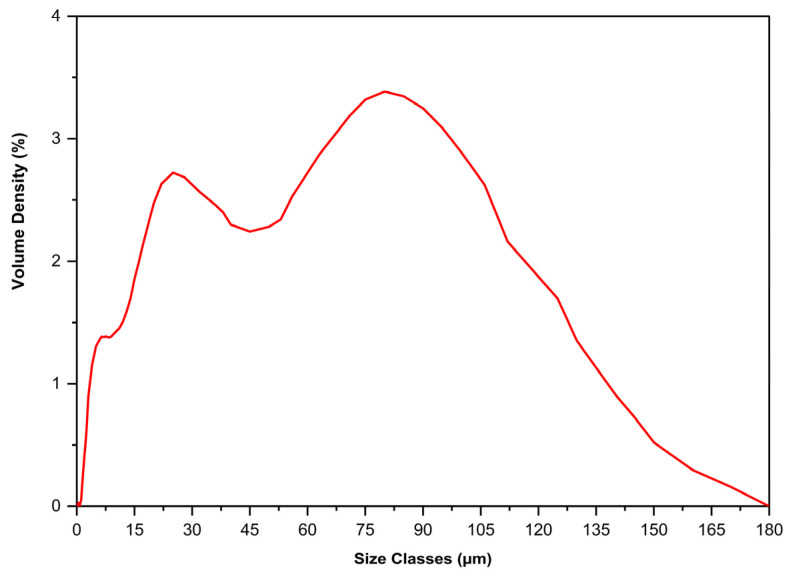
Particle size distribution of CSW.

The particle size distribution of the CSW powder was evaluated using laser diffraction analysis with a Mastersizer 2000 analyzer (Malvern Panalytical, Malvern, UK) in accordance with ISO-13320 standard [40]. The characteristic particle diameters d_10_, d_50_, and d_90_ were determined to be 3.74 µm, 28.90 µm, and 90.46 µm, respectively, as presented in Figure 3.

**Figure 4:**
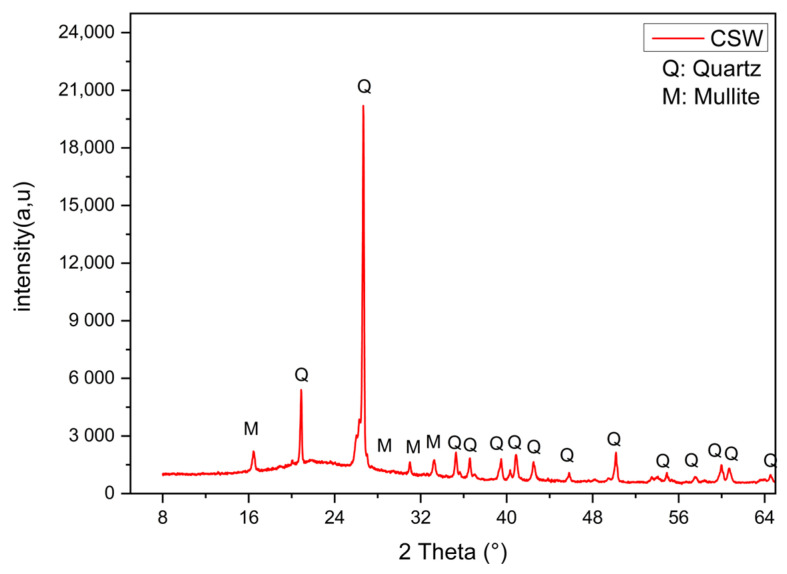
X-ray diffraction pattern of CSW powder.

Mineralogical characterization was carried out using X-ray diffraction (XRD) analysis with a (Bruker, Karlsdorf, Germany) D4 ENDEAVOR model diffractometer employing Cu Kα radiation over a 2θ range of 0–65°, a step size of 0.02° 2θ, and a count time of 0.1 s per step; the voltage on the X-ray tube was 40 kV and the current 35 mA. Phase identification was performed using X’Pert HighScore Plus software V5.3a in combination with the ICDD PDF-2 database. The XRD pattern presented in Figure 4 indicates that quartz (SiO_2_) and mullite (Al_4.44_Si_1.56_O_9.78_) were the predominant crystalline phases in the CSW, in agreement with previous studies [14]. In addition, the broad diffuse hump observed between 15° and 30° (2θ) suggests the presence of an amorphous or poorly crystalline phase.

### 3.2. Workability of Fresh Mortars

**Figure 5:**
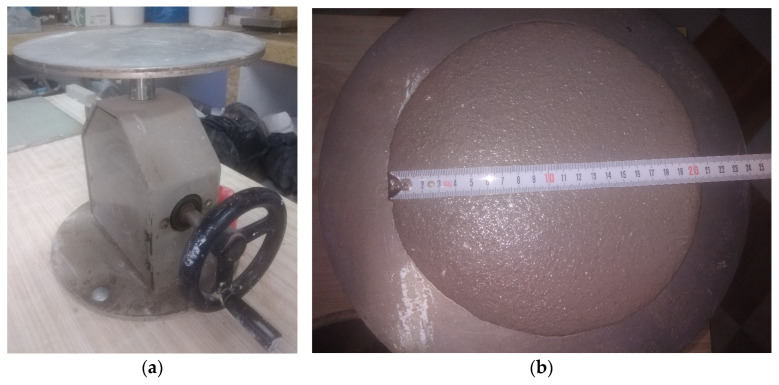
Mini cone flow table test: (**a**) The used tool; (**b**) The measurement.

The flowability of the geopolymer mortars was assessed using the slump flow test in accordance with ASTM C143715 [41], as illustrated in Figure 5. The corresponding flow diameters are reported in Table 3 for the mortar composition.

A flow diameter of 21 cm was obtained for the geopolymer mortar incorporating 100% ceramic sanitaryware waste (CSW) and activated with 10 Mol NaOH at a liquid-to-solid (L/S) ratio of 0.45. The result indicated moderate workability, consistent with values typically reported for geopolymer mortars [42]. The measured flowability confirms that the selected activator concentration and L/S ratio provide a suitable consistency for casting and handling.

Comparable flow ranges have been documented in the literature under similar activation conditions. For instance, Rezzoug et al. [6] reported flow diameters between 18 and 21 cm for CSW-based mortars activated with 10–16 M NaOH at an L/S ratio of 0.45. In contrast, Atabey [16] observed lower flow values, ranging from 10.3 to 11.5 cm at an L/S ratio of 0.45 and from 15.8 to 19.2 cm at an L/S ratio of 0.50, highlighting the sensitivity of fresh-state behavior to mixture proportions even under comparable conditions.

To further explain these differences, the influence of mix design parameters on flowability has been widely reported. Previous works by [43] stated that increasing the water-to-binder ratio enhances fluidity, whereas reducing the binder-to-sand ratio decreases workability due to the angular nature of sand particles, which increases internal friction and limits paste mobility. In the present study, the selected L/S ratio of 0.45 provides a balanced mixture, ensuring adequate flowability without segregation.

Beyond mix proportions, the variability in flow behavior can also be attributed to the intrinsic characteristics of ceramic sanitaryware waste (CSW), particularly its mineralogical composition, particle fineness, and morphology, which collectively govern dissolution kinetics and particle packing in the fresh state [44]. As a result of this, differences in CSW reactivity and variations in mixing procedure may further contribute to the observed variability in fresh-state behavior [45].

### 3.3. Mechanical Performance

**Figure 6:**
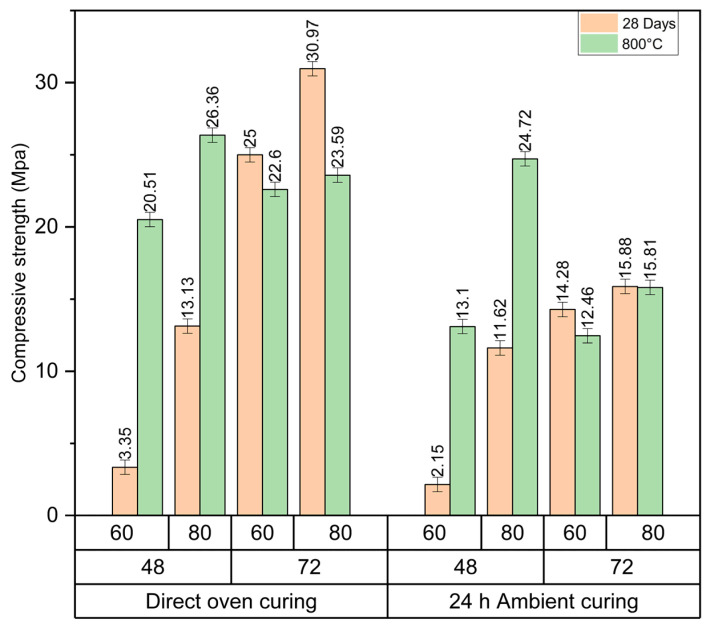
Compressive strength of geopolymer mortar samples only cured and after exposed to high temperatures.

Figure 6 presents the compressive strength results of the geopolymer mortars evaluated after 28 days. The compressive strength results clearly demonstrate that the curing regime exerts a governing influence on both mechanical performance and residual mechanical performance after thermal exposure of CSW-based geopolymer mortars. Specimens subjected to direct thermal curing consistently outperformed those exposed to delayed curing. For example, at 60 °C for 48 h, direct curing yielded a compressive strength of 3.35 MPa, compared to 2.15 MPa for delayed curing. A similar trend was observed at 80 °C, where strengths of 13.13 MPa and 11.62 MPa were recorded for direct and delayed curing, respectively. This enhancement can be attributed to immediate thermal activation, which accelerates the dissolution of aluminosilicate species and promotes continuous polycondensation from the earliest stages, leading to the formation of a denser and more cohesive geopolymer network as reported by [46]. In contrast, the delayed curing appears to have limited the effectiveness of early-stage thermal activation under the investigated conditions.

An increase in curing temperature from 60 °C to 80 °C significantly enhanced compressive strength for both curing regimes, highlighting the critical role of thermal energy in governing reaction kinetics [34]. Under direct curing, strength increased from 3.35 MPa to 13.13 MPa at 48 h and from 25.0 MPa to 30.97 MPa at 72 h. Similarly, delayed-cured specimens exhibited improvements from 2.15 MPa to 11.62 MPa at 48 h and from 14.28 MPa to 15.88 MPa at 72 h. The positive influence of elevated curing temperature was consistent with previous studies on ceramic waste-based geopolymers, where enhanced dissolution of reactive phases and accelerated polycondensation were observed under moderate thermal curing conditions [47].

Curing duration also had a pronounced effect on strength development, with systematic increases observed when the heat curing was extended from 48 h to 72 h. This effect was particularly significant under direct curing conditions, suggesting sustained reaction progress and continued geopolymer gel development during prolonged curing [33]. These findings were supported by the SEM observations, where directly cured specimens exhibited a more homogeneous microstructure with fewer visible voids and unreacted particles. In contrast, indirectly cured specimens showed a more heterogeneous microstructure, which was consistent with their lower compressive strength. Conversely, delayed-cured mortars exhibited more limited strength gains, reflecting restricted reaction kinetics during the initial ambient curing stage.

The residual compressive strength after exposure to 800 °C further confirms the influence of curing conditions on the thermal behavior of the investigated systems. At 60 °C for 48 h, residual strength increased from 13.10 MPa for delayed curing to 20.51 MPa for direct curing, while at 80 °C for 48 h, values increased from 24.72 MPa to 26.36 MPa. The highest residual strength of 26.36 MPa was obtained for specimens directly cured at 80 °C for 48 h, followed by those cured for 72 h of 23.59 MPa. The comparatively high residual strengths observed after thermal exposure may be related to inherent high-temperature resistance of the ceramic sanitaryware waste, particularly due to the persistence of quartz and mullite phases identified by XRD analysis. Similar behavior was reported by Celikten et al. [6,48], who highlighted the ability of ceramic-based geopolymer systems to retain significant mechanical integrity after exposure to elevated temperatures. Furthermore, the presence of thermally stable crystalline phases, such as quartz and mullite inherited from residual parent ceramic material, in conjunction with partial sintering effects at elevated temperatures, may contributed to improved strength retention as reported by [49,50].

Although longer curing durations of 72 h improved compressive strength before thermal exposure, a more noticeable reduction in residual strength was observed after exposure to 800 °C compared with specimens cured for 48 h, which exhibited better strength retention. This behavior may be associated with increased water evaporation during prolonged thermal curing, leading to microstructural shrinkage and the development of internal stresses that facilitate crack propagation under elevated temperatures [51,52].

### 3.4. Microstructural Analysis

#### 3.4.1. Crystalline Phase

**Figure 7:**
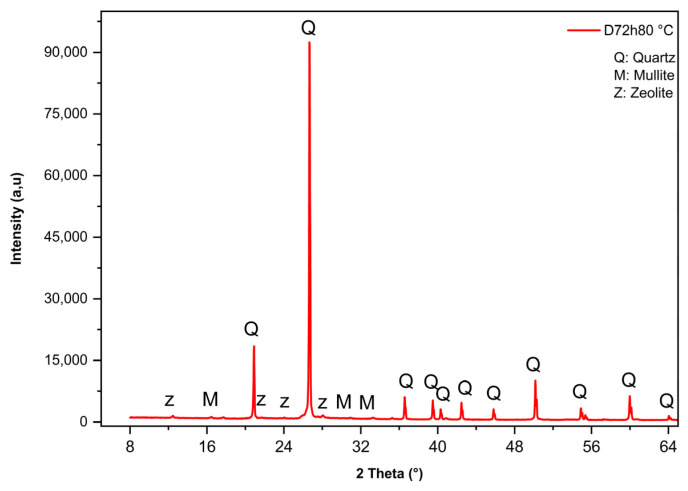
XRD pattern of CSW-based geopolymer mortar after direct curing at 80 °C for 72 h.

Figure 7 shows the XRD patterns of geopolymer mortar specimens subjected to direct thermal curing at 80 °C for 72 h, analyzed to complement the SEM-based microstructural observations.

The diffraction patterns indicate that quartz and mullite were the predominant crystalline phases. Mullite was mainly associated with the ceramic sanitaryware waste (CSW) precursor, whereas quartz originates from both the CSW and the standard silica sand used as fine aggregate. In addition, a minor crystalline phase was identified, suggesting the formation zeolitic phase during the geopolymerization process as shown in MEB/EDS image, and similar observations were reported by [6,16,42].

The persistence of mullite diffraction peaks indicates that this phase underwent only partial dissolution during alkaline activation [16]. However, the reduction in peak intensity relative to the raw CSW powder suggests that a fraction of the mullite participated in the reaction, releasing reactive aluminosilicate species that contributed to the geopolymerization.

The presence of zeolitic phases may indicate the development of crystalline aluminosilicate species within the geopolymer-based CSW [49]. Such phases are commonly reported in alkali-activated systems exposed to elevated curing temperatures and are generally associated with post-geopolymerization structural evolution as reported by [53]. Their detection suggests that curing at 80 °C for 72 h may have contributed to localized structural changes within the predominantly amorphous matrix.

Furthermore, the XRD pattern exhibits a broad diffuse characteristic of an amorphous aluminosilicate gel, which may show that the main binding phase of the geopolymer matrix was largely amorphous despite the presence of minor crystalline products [49]. This observation, together with the partial persistence of mullite, was consistent with the SEM results.

These microstructural features were in agreement with the findings of Liu et al. [24], who reported that geopolymerization was mainly governed by the dissolution of the amorphous aluminosilicate gel network.

#### 3.4.2. Scanning Electron Microscopy (SEM/EDS)

**Figure 8:**
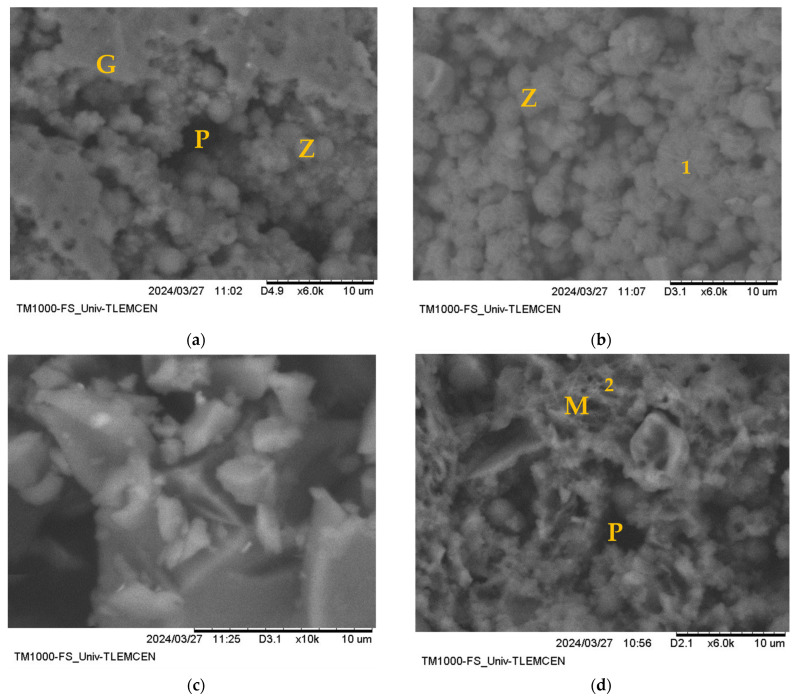
SEM analysis of: (**a**) Only cured D72h80 °C; (**b**) Only cured ID72h80 °C; (**c**) After heated D72h80 °C; (**d**) After heated ID72h80 °C. (G: Gel, M: mullite, Z: Zeolite, P: Pore, 1: Spot 1, 2: Spot 2).

The SEM micrographs presented in Figure 8 provide insights into the microstructural evolution of the geopolymer systems under different curing conditions and after thermal exposure, in relation to the observed mechanical performance.

After curing, noticeable differences in morphology were observed between the two curing regimes. Specimens subjected to direct thermal curing exhibit a more uniform and compact microstructure with fewer visible voids (Figure 8a), whereas indirectly cured specimens show a more heterogeneous morphology characterized by unreacted particles and a higher porosity level (Figure 8b). These microstructural differences were consistent with the variations observed in compressive strength, where directly cured specimens present higher mechanical performance than indirectly cured ones.

**Figure 9:**
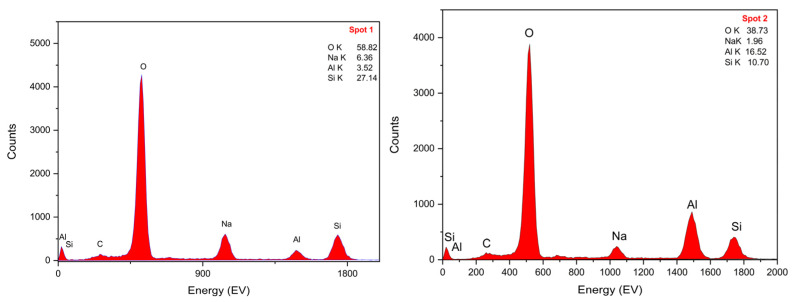
EDS analysis of SEM spot 1 and spot 2.

To further support the microstructural interpretation, EDS spot analyses were performed on selected regions. EDS spot 1 in Figure 9 reveals Na–Si-rich zones with compositions consistent with sodium aluminosilicate hydrate (N–A–S–H)-type gel, which may also be associated with the possible presence of zeolitic-type phases, as suggested by similar observations reported in previous studies [49]. EDS spot 2 indicated chemical compositions compatible with mullite-related phases. These findings were in agreement with previous studies on ceramic waste-based geopolymers [6,54,55].

These observations were also consistent with the XRD results, which indicate quartz and mullite as the main crystalline phases. Accordingly, the possible formation of zeolite was suggested.

After thermal exposure at 800 °C, directly cured specimens exhibit limited microcrack development together with localized microstructural modifications, while the mullite phase remains detectable, as shown in Figure 8c and Figure 9 (spot 2). Similar observations were reported in the authors’ previous study [6] and also by [16], whereas indirectly cured specimens exhibit more pronounced cracking and a more disrupted microstructure (Figure 8d). These changes were consistent with the reduction in residual compressive strength.

## 4. Conclusions, Limitations, and Future Works

This study investigated the influence of direct and delayed thermal curing on the mechanical performance, residual strength after high-temperature exposure, and microstructural behavior of geopolymer mortars based on ceramic sanitaryware waste (CSW).

The results showed that curing conditions significantly affect mechanical development behavior. Direct thermal curing consistently resulted in higher compressive strength compared with delayed curing, reaching a maximum value of 30.97 MPa at 80 °C for 72 h. An increase in curing temperature from 60 °C to 80 °C, as well as a prolonged curing duration from 48 h to 72 h, generally enhanced the compressive strength for both curing regimes, indicating the beneficial role of prolonged thermal activation in improving early-age performance.

After exposure to 800 °C, directly cured specimens maintained higher residual compressive strength than delayed-cured specimens. However, the results also indicated that curing duration influenced the thermal response, as specimens cured for 48 h exhibited slightly better residual strength retention compared with those cured for 72 h. This suggests that prolonged thermal curing may not always be favorable.

SEM/EDS and XRD analyses confirmed differences in microstructural condition between curing regimes and supported the mechanical results, showing a more coherent residual structure in directly cured specimens and higher microstructural discontinuities in delayed-cured ones.

In summary, CSW has been shown to be a promising precursor for geopolymer mortar production, particularly when optimized thermal curing conditions were applied. However, the applicability of the obtained results was mainly relevant to systems designed for conventional casting applications requiring external thermal curing, rather than ambient-cured or early-strength-demanding applications.

The present study was limited to a single activator concentration and a fixed liquid-to-solid ratio, and did not investigate ambient curing conditions without thermal activation. In addition, detailed chemical and kinetic analyses were not performed. Therefore, future research should focus on investigating the interaction between curing conditions and mixture composition using advanced characterization techniques. Further studies are also recommended to explore the potential application of CSW-based geopolymers in advanced manufacturing techniques such as 3D printing, as well as the use of blended aluminosilicate precursor systems and the evaluation of long-term durability and sustainability performance under real environmental conditions.

## Figures and Tables

**Table 1:** Mortar mixtures code.

Curing Method	Molarity	Curing Duration	Mixture Code	Curing Temperature (°C)	L/S	CSW (g)	Sand (g)	NaOH Pellets (g)	Distilled Water (g)
Direct oven curing	10 M	48 h	D48h60 °C	60	0.45	450	1350	202.5	82.65
			D48h80 °C	80	0.45	450	1350	202.5	82.65
		72 h	D72h60 °C	60	0.45	450	1350	202.5	82.65
			D72h80 °C	80	0.45	450	1350	202.5	82.65
24 h ambient before curing	10 M	48 h	ID48h60 °C	60	0.45	450	1350	202.5	82.65
			ID48h80 °C	80	0.45	450	1350	202.5	82.65
		72 h	ID72h60 °C	60	0.45	450	1350	202.5	82.65
			ID72h80 °C	80	0.45	450	1350	202.5	82.65

**Table 2:** Chemical composition of ceramic sanitaryware waste (CSW) determined by XRF.

Oxides (%)	CSW
SiO_2_	69.56
Al_2_O_3_	22.61
CaO	0.94
FeO	0.99
K_2_O	1.45
MgO	0.14
Na_2_O	0.41
TiO_2_	0.44
LOI *	1.1
Specific gravity	2.6
Blaine Surface (cm^2^/g)	3500

* LOI: Loss on ignition.

**Table 3:** Flow diameter for the mixture.

NaOH Concentration (mol)	Ratio L/S	Flow Diameter (cm)
10	0.45	21

## Data Availability

The data presented in this study are fully within the article.
